# Redox Imbalance and Mitochondrial Release of Apoptogenic Factors at the Forefront of the Antitumor Action of Mango Peel Extract

**DOI:** 10.3390/molecules26144328

**Published:** 2021-07-17

**Authors:** Valentina Lo Galbo, Marianna Lauricella, Michela Giuliano, Sonia Emanuele, Daniela Carlisi, Giuseppe Calvaruso, Anna De Blasio, Diana Di Liberto, Antonella D’Anneo

**Affiliations:** 1 Department of Biological, Chemical and Pharmaceutical Sciences and Technologies (STEBICEF), Laboratory of Biochemistry, University of Palermo, 90127 Palermo, Italy; valentina.logalbo@unipa.it (V.L.G.); michela.giuliano@unipa.it (M.G.); giuseppe.calvaruso@unipa.it (G.C.); anna.deblasio@unipa.it (A.D.B.); 2 Department of Biomedicine, Neurosciences and Advanced Diagnostics (BIND), Institute of Biochemistry, University of Palermo, 90127 Palermo, Italy; sonia.emanuele@unipa.it (S.E.); daniela.carlisi@unipa.it (D.C.); 3 Department of Biomedicine, Neurosciences and Advanced Diagnostics (BIND), CLADIBIOR, University of Palermo, 90127 Palermo, Italy; diana.diliberto@unipa.it

**Keywords:** phytochemicals, mitochondria injury, Bcl-2 family proteins, mitochondrial apoptogenic proteins

## Abstract

Today, an improved understanding of cancer cell response to cellular stress has become more necessary. Indeed, targeting the intracellular pro-oxidant/antioxidant balance triggering the tumor commitment to cell demise could represent an advantageous strategy to develop cancer-tailored therapies. In this scenario, the present study shows how the peel extract of mango—a tropical fruit rich in phytochemicals with nutraceutical properties—can affect the cell viability of three colon cancer cell lines (HT29, Caco-2 and HCT116), inducing an imbalance of cellular redox responses. By using hydro-alcoholic mango peel extract (MPE), we observed a consistent decline in thiol group content, which was accompanied by upregulation of MnSOD—a mitochondrial scavenger enzyme that modulates the cellular response against oxidative damage. Such an effect was the consequence of an early production of mitochondrial superoxide anions that appeared after just 30 min of exposure of colon cancer cells to MPE. The effect was accompanied by mitochondrial injury, consisting of the dissipation of mitochondrial membrane potential and a decrease in the level of proteins localized in the mitochondrial membrane—such as voltage-dependent anion-selective channel (VDAC1), mitofilin, and some members of Bcl-2 family proteins (Mcl-1, Bcl-2 and Bcl-X_L_)—with the mitochondrial release of apoptogenic factors (cytochrome C and AIF). The analysis of the cytotoxic effects exerted by the different constituents of MPE (gallic acid, mangiferin, citric acid, quinic acid, pentagalloyl glucose, and methyl gallate) allowed us to identify those phytochemicals responsible for the observed anticancer effects, sustaining their future employment as chemopreventive or therapeutic agents.

## 1. Introduction

The maintenance of redox homeostasis represents a strategic aim of cancer cells [1]. The alteration of this balance leads to the generation of reactive oxygen species (ROS) which, as byproduct of aerobic metabolism, can act in two opposite ways in neoplastic cells: either fostering the cancer phenotype by enabling specific hallmarks, such as uncontrolled cell proliferation, migration, and survival; or triggering cell death following overpowered production [2].

To cope with changes in redox homeostasis, cancer cells have developed adaptive scavenging mechanisms to check the increased oxidative stress conditions. Defense systems, such as antioxidant enzymes or other oxygen scavenging pathways, are highly active in cancer cells, such that their tolerance threshold for oxidative stress is higher than that of normal cells [3]. The increase in ROS is decisive to individuate their cellular effect, indicated as the hormetic role of ROS. Recently, the terms “oxidative distress” and “oxidative eustress” have been coined. In particular, eustress indicates the role of physiological doses of ROS (in particular, hydrogen peroxide) as intracellular signaling molecules, while distress is related to general molecular damage [4]. Moreover, sometimes, exceeding the threshold induced by exogenous molecules or intracellular imbalance can trigger programmed cell death. In this regard, many studies have proven that high levels of ROS can make cancer cells more susceptible to the devastating action of additional ROS. Thus, a possible strategy that has been considered in cancer research is to destroy cellular antioxidant scavenging systems and enhance ROS generation [5].

It is widely known that the main source of ROS in cells is represented by the mitochondrial electron transport chain [6,7]. An increase in ROS levels may result from an accelerated metabolism or a mitochondrial dysfunction, as well as from treatment with different antitumor drugs. Other important sources of intracellular ROS are represented by NADPH oxidase and xanthine oxidase activities. In addition, the generation of reactive nitrogen species can also contribute to the increase in mitochondrial ROS production [8].

The outcome of increased ROS levels—either promoting tumor proliferation or, conversely, tumor growth suppression and cell death induction—is related to the ability of mitochondria to release the apoptogenic factors, such as the pro-apoptotic members of the Bcl-2 family, or AIF and cytochrome C.

An interesting bidirectional relationship exists between the mitochondria and the nucleus [9]. Following nuclear DNA damage promoted by different inducers (e.g., ultraviolet light, DNA-damaging drugs, etc.), cells respond by arresting the cell cycle, inducing DNA repair or, ultimately, inducing apoptotic cell death. In this case, the mitochondria play a central role; in particular, they switch from the “powerhouse” of the cell to the “headquarters” of cell death, actively participating in a canonical intrinsic apoptotic pathway.

On the other hand, the release of mitochondrial ROS influences nuclear events and induces the oxidation of nucleoside bases, which compromises DNA stability and interferes with DNA replication [10].

In recent years, particular interest has been paid to the anticancer potential of natural products derived from fruits, plants, or chemically modified phytocompounds for use as new resources that can be exploited to pursue new therapeutic approaches to cancer. 

Recently, we focused on the anticancer activity of *Mangifera indica L.* fruit. This plant is cultivated all over the world and, although its crops predominate in tropical climates [11], their settlement is also widespread in subtropical areas. Among these are the southern regions of the Italian Peninsula, such as Sicily [12,13,14], where the favorable pedoclimatic conditions have allowed the cultivation of mangoes in the past decade. 

The great nutraceutical value of mango fruit lies in its content of macronutrients (such as carbohydrates and lipids), micronutrients (vitamins and minerals), and phytocompounds (polyphenols) [15]. In recent years, several investigations were carried out using the different fractions (peel, pulp, and seed) and phytocompounds belonging to the fruit [16,17,18]. Some findings highlighted cytotoxic and antitumor activity of *Mangifera indica L.* extracts in vitro and in vivo on different carcinoma cell lines. In particular, Noratto et al. [19] demonstrated that secondary metabolites from plants—such as polyphenols extracted from pulp of many different mango cultivars—possess anticancer activity against several cellular types, such as MDA-MB231 breast cancer cells, MOLT-4 leukemia cells, SW-480 colon cancer cells, and A-549 lung cancer cells. Such an effect was ascribed to the induction of pro-apoptotic factors and cell cycle arrest, and downregulation of reactive oxygen species [19]. The antitumor potential of mango fruit was also confirmed in in vivo studies. Banerjee’s findings revealed that mango polyphenols counteract breast cancer growth in murine xenografts by suppressing the PI3K/Akt pathway, downregulating HIF and VEGF mRNAs, and modulating miR126 [20].

In line with these findings, in a recent paper, we demonstrated that hydro-alcoholic mango peel extract (MPE) from Sicilian mango fruit shows remarkable antitumor effects in colon cancer cells. The effect was accompanied by a DNA damage response consisting of the early phosphorylation of histone 2AX (γH2Ax) and ATM kinase activation, as well as p53 upregulation [21]. Moreover, we observed an increase in apoptotic marker levels associated with ROS production.

In this paper, we analyze the role of the mitochondria in MPE-induced cell death, in an attempt to individuate the specific crosstalk between mitochondrial events and nuclear genotoxic stress. Moreover, we screened the cytotoxic effects induced by single phytochemicals, in order to discover the most represented bioactive components of MPE.

## 2. Results

### 2.1. MPE Treatment Promotes a Dramatic Decrease in Thiol Group Content, Changes in MnSOD Enzyme, and Mitochondrial Superoxide Anion Generation

Our previous studies provided evidence that Sicilian mango peel extract (MPE) markedly reduces colon cancer cell viability via apoptotic cell demise associated with genotoxic events, involving a precocious generation of ROS, H2Ax phosphorylation (γH2Ax), and DNA fragmentation. It is interesting to note that the inhibitory effect of MPE only occurs in colon cancer cells, while it turns out to be ineffective in human dermal fibroblasts [21]. Therefore, in order to better define the antitumor action of the extract we undertook a study aimed at exploring whether MPE could affect the functionality of the mitochondria—the powerhouse of the cell. 

**Figure 1:**
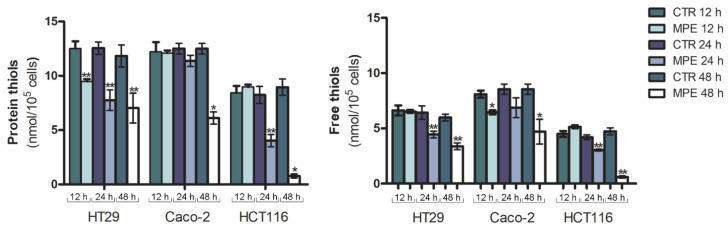
MPE treatment promotes the depletion of thiol group content. For these experiments, 10^6^ cells/condition were incubated for different times (12, 24, and 48 h) with 360 μg/mL MPE, and then the amount of protein and free thiol groups was assessed, as reported in the Materials and Methods section. The values, expressed as nmol/10^5^ cells, are the mean of three independent experiments ± S.E. (*) *p* < 0.05 and (**) *p* < 0.01 versus untreated control.

With this in mind, we firstly focused on the analysis of the redox balance system of tumor cells—and in particular on the non-enzymatic antioxidants known as thiols, as it is known that the maintenance of thiol status, as well as thiol/disulfide balance, represents one of the most important aims of the cell. Total thiols—represented by both intracellular and extracellular thiols, as well as thiols bound to proteins—are part of the protective system to counteract oxidative injury. Changes in their status have been shown to coincide with the pathogenesis of stress-associated disorders [22]. To this purpose, the content of free and protein thiols was assessed via Ellman’s method after different times of incubation (12, 24, and 48 h) of cells in the presence of MPE. Analyzing the thiol content of treated cells, we found that the extract promoted a remarkable depletion of both protein and free thiol groups. Such a decrease was clearly visible in all three colon cancer cell lines after 24 h of incubation, and was particularly pronounced after 48 h of treatment (Figure 1), when the content of both protein and free thiols reached a value close to zero in HCT116 cells. 

**Figure 2:**
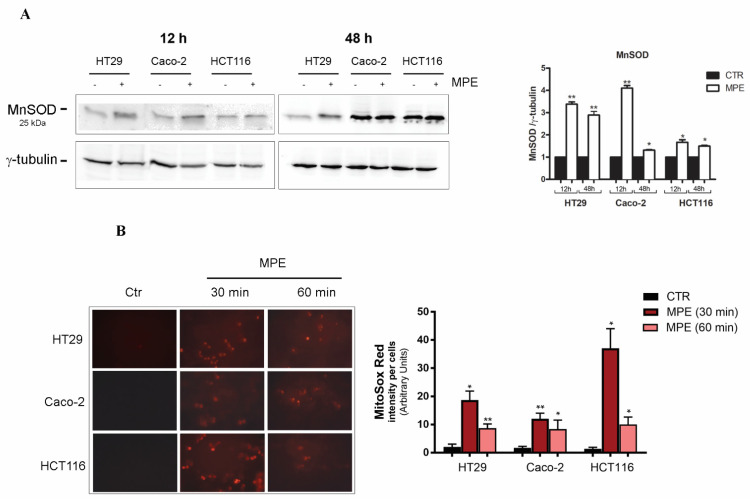
MPE treatment promotes the upregulation of MnSOD and mitochondrial superoxide anion generation. (**A**) Western blot analysis of MnSOD in colon cancer cells treated with 360 μg/mL MPE for 12 h and 48 h. Analyses were performed as reported in the Materials and Methods section. The correct protein loading was ascertained by immunoblotting for γ-tubulin. A representative blot of three independent experiments and densitometry analysis histograms are reported. (*) *p* < 0.05 and (**) *p* < 0.01 versus untreated control. (**B**) Micrographs of fluorescence microscopy showing mitochondrial superoxide anion generation in colon cancer cells incubated in the absence or presence of 360 μg/mL MPE for the indicated times. A bar chart representing the densitometric fold difference in MitoSOX-Red-positive cells is reported in the right panel. Pictures were taken using a Leica fluorescent microscope using a rhodamine filter. (*) *p* < 0.05 and (**) *p* < 0.01 versus untreated control.

As evidenced in Figure 2A, the depletion of thiol group content, as a consequence of cell response to stress, was accompanied, after just 12 h of treatment, by upregulation of manganese superoxide dismutase (MnSOD)—a known oxy-radical scavenger mitochondrial enzyme that can be activated in cellular response to oxidative damage.

In order to ascertain whether changes in thiol group content could be ascribed to stress-mediated mitochondrial damage, we incubated MPE-treated cells in the presence of MitoSOX Red— a cationic triphenylphosphonium substituent that specifically targets the mitochondria, and produces red fluorescence when it is oxidized by superoxide anions. As depicted in Figure 2B, in accordance with our previous studies demonstrating precocious ROS production in colon cancer cells treated with MPE [21], the exposure to the extract promoted an early generation of superoxide anions that reached a maximum effect after just 30 min of treatment. 

### 2.2. MPE Causes Mitochondrial Membrane Potential Dissipation in Colon Cancer cells

After analyzing the effects of MPE treatment on cellular redox balance and mitochondrial superoxide anion generation, we explored whether the extract could exert a cytotoxic effect on mitochondrial functionality. To this purpose, we investigated the dissipation of mitochondrial membrane potential (Δψm) in colon cancer cells using JC1 dye—a membrane-permeant fluorescent probe usually applied to establish mitochondrial health state during apoptotic cell death. 

**Figure 3:**
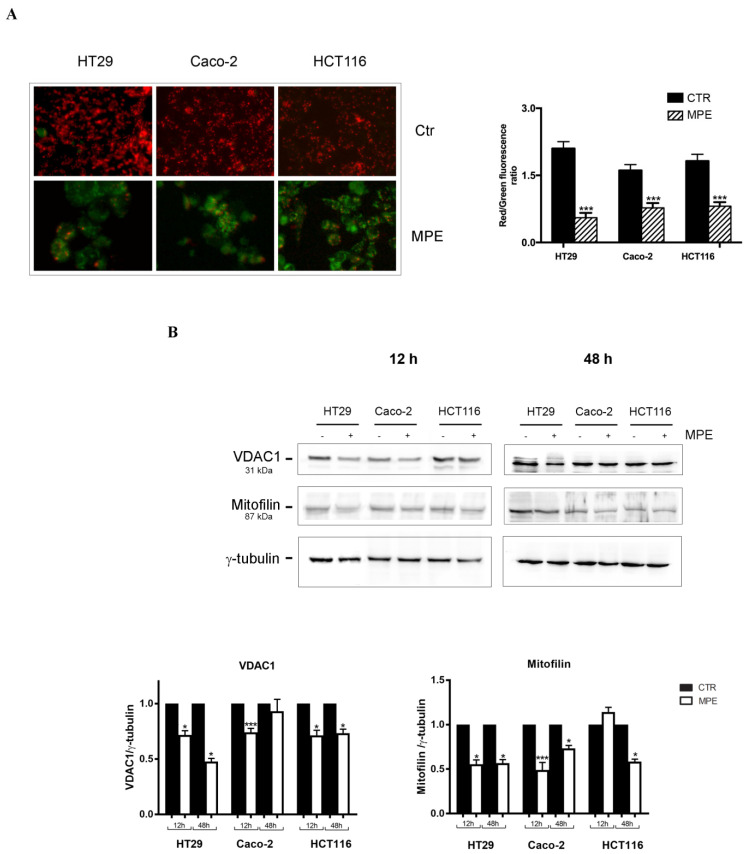
MPE treatment causes dissipation of mitochondrial membrane potential (Δψm) and changes in mitochondria-associated proteins. (**A**) JC-1 staining on colon cancer cells untreated and treated with 360 μg/mL MPE for 48 h. Red fluorescence, suggestive of a high mitochondrial membrane potential (preserved Δψm favors JC-1 aggregates), was observed in almost all untreated cells, whereas green fluorescent signals, indicative of low mitochondrial membrane potential (depolarization favors JC1 monomers), occurred in MPE-treated colon cancer cells. Merged images were obtained as reported in the Materials and Methods section, and taken at 200x magnification (original). Quantification of green and red fluorescent cells (expressed in percentages) is reported in the bar chart. Data were from three independent experiments, each with 50 counted cells. The data are reported as mean ± SD. (***) *p* < 0.001 versus untreated control. (**B**) Western blot analysis of the mitochondria-localized proteins. Cells were treated for 12 h and 48 h, then total cell extracts were subjected to Western blot analysis and tested for VDAC1 and mitofilin. The correct loading was checked for γ-tubulin protein. The densitometric analysis data reported are the mean of results obtained via three separate experiments that were normalized to the γ-tubulin protein. (*) *p* < 0.05 and (***) *p* < 0.001 versus untreated control.

As shown in Figure 3A, our data provided evidence for a red fluorescence (J-aggregates) in all untreated colon cancer cells, while the prevalence of a strong green fluorescence (monomeric form)—indicative of the dissipation of mitochondrial membrane potential—was observed in most MPE-treated cells. To corroborate these data, the red/green ratio was calculated. The ratio decreased in treated cells by 74% in HT29, 52% in Caco-2, and 56% in HCT116 cells. These findings demonstrate that MPE causes mitochondrial damage via the dissipation of mitochondrial membrane potential—an event indicative of the pro-apoptotic activity of MPE.

Mitochondrial injury was also monitored via analyses conducted on proteins involved in the maintenance of mitochondrial integrity, such as VDAC1 (voltage-dependent anion-selective channel 1) and mitofilin. As known, VDAC1 represents a multifunctional mitochondrial factor involved in the production of an ion channel in the outer mitochondrial membrane, where it monitors the flux exchange of metabolites between the mitochondria and the cytosol. VDAC1 has been also demonstrated to be a gatekeeper of apoptotic demise mediated by the mitochondria [23]. 

As evidenced in Figure 3B, the loss of mitochondrial membrane potential was reflected by VDAC1 downregulation that, after just 12 h of treatment, was more consistent in HT29 and Caco-2 cells than in HCT116 cells. Particularly interesting was the effect of MPE on mitofilin—a mitochondrial morphology-shaping factor operating in the inner mitochondrial membrane, where it regulates the morphology of mitochondrial crests as well as their remodeling, thus favoring the crest junction in the inner mitochondrial membrane [24]. In our experiments, the level of mitofilin was downregulated in MPE-treated cells vs. the control, suggesting that the cytotoxic effects of this fraction of mango, in association with VDAC1 downregulation and superoxide anion production, can be ascribed to mitochondrial damage. 

### 2.3. MPE Treatment Affects the Level of Some Antiapoptotic Members of the Bcl-2 Family

In order to further understand the possible mechanisms of MPE-induced cytotoxicity on colon cancer cells, we investigated the effects of the extract on the levels of Bcl-2 family members—examples of evolutionarily well-conserved proteins that, in relationship to the specific family member, can antagonize one another either in survival or in death signals, and govern mitochondrial dynamics. These factors anchor to the outer mitochondrial membrane, and, regulating its permeabilization, can determine cell commitment to apoptotic demise. However, beyond these aspects, a new paradigm of the non-ordinary cell death roles of these protein family members, not strictly related to cell demise, has also emerged [25]. In this regard, it has been demonstrated that both Bcl-2 and Bcl-X_L_ can also act as crucial regulators of other important cellular processes, such as proliferation, autophagy, tumor progression, DNA repair, and angiogenesis [26]. 

**Figure 4:**
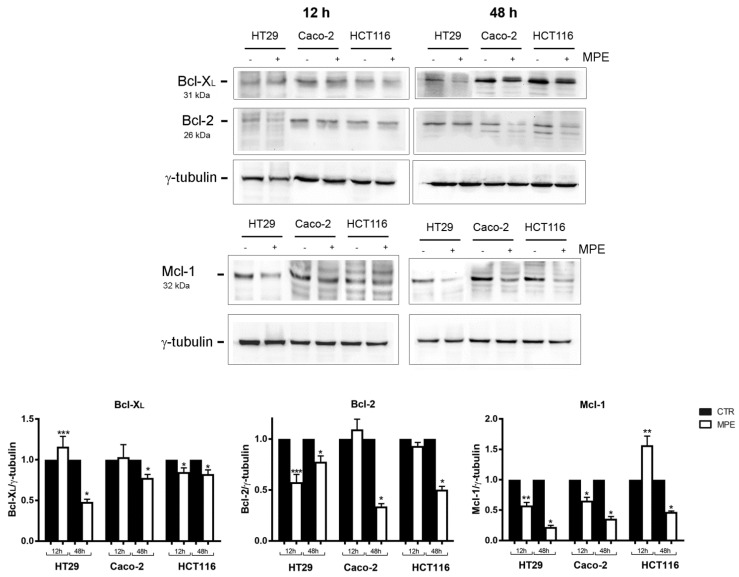
MPE treatment downregulates antiapoptotic Bcl-2 family members in colon cancer cells. After 12 h and 48 h of incubation in the absence or presence of 360 μg/ml MPE, colon cell lines (HT29, Caco-2, and HCT116) were lysed and proteins were analyzed via Western blot, using specific antibodies for the proteins of interest. The blot of the γ-tubulin is also reported as a control of a homogeneous loading. The densitometry data were acquired using Quantity One software. For each set of data, the mean value of results obtained in three independent experiments was calculated and normalized to the housekeeping γ-tubulin protein. (*) *p* < 0.05, (**) *p* < 0.01, and (***) *p* < 0.001 versus untreated control.

As shown in Figure 4, the addition of MPE reduced the content of both Bcl-2 and Bcl-X_L_ proteins in all three colon cancer cell lines. Another particularly interesting effect was that of the MPE on Mcl-1—a prominent Bcl-2 family member with an antiapoptotic role, whose overexpression and amplification represent events frequently occurring in human cancers [27]. Some studies have also emphasized it as an essential player in stabilizing mitochondrial functions, such as ATP generation, mitochondrial membrane potential, and oxygen consumption rate. We found that colon cancer cells—and in particular Caco-2 and HCT116 cells—express high levels of Mcl-1 that were dramatically downregulated by MPE exposure for 48 h. Such an effect was consistent with the findings of Tong et al., who suggested a pivotal role of Mcl-1 in modulating the resistance of colon cancer cells to targeted chemotherapeutics [27]. 

### 2.4. MPE-Mediated Mitochondrial Injury Promotes the Mitochondrial Release of the Apoptogenic Proteins Cytochrome C and AIF

It is well known that the mitochondria rely on an arsenal of apoptogenic factors such as SMAC/DIABLO, endonuclease G, apoptosis-inducing factor (AIF), and cytochrome C, which can be released following changes in mitochondrial permeability [28]. In order to explore whether mitochondrial injury caused by MPE could promote the mitochondrial release of apoptogenic factors, we analyzed the distribution of cytochrome C and AIF in the mitochondrial and cytosolic fractions.

**Figure 5:**
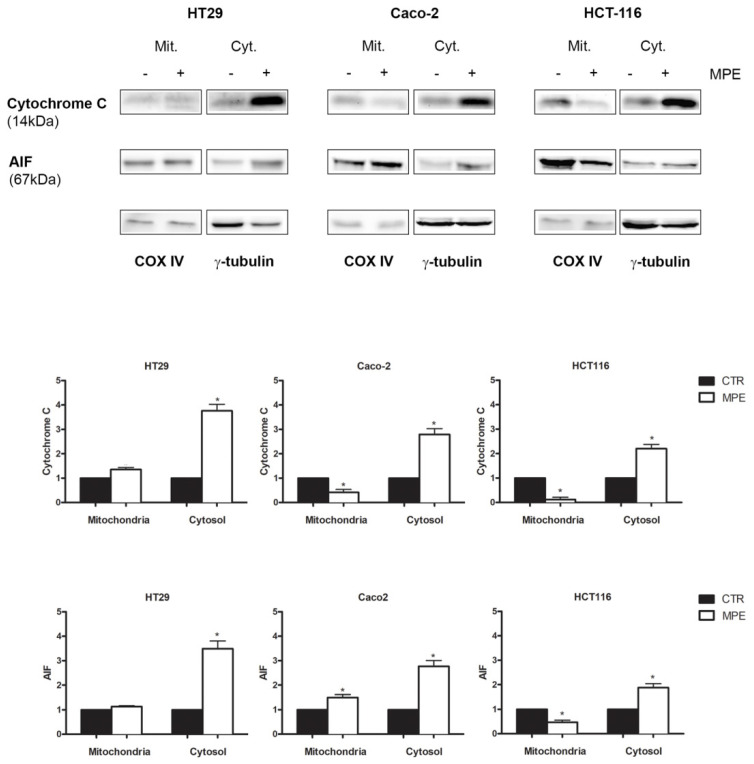
MPE exerts its cytotoxic action through the mitochondrial release of apoptogenic factors. HT29, Caco-2, and HCT116 colon cancer cells (1.5 × 10^7^) were exposed to MPE treatment for 48 h and then were scraped and submitted to subcellular fractionation, as described in the Materials and Methods section. Thirty micrograms of proteins of each fraction were placed in a polyacrylamide gel and submitted to Western blot analysis. As loading controls for the mitochondrial and cytosolic fractions, COX IV and γ–tubulin were used, respectively. (*) *p* < 0.05 versus untreated control.

As reported in Figure 5, a 3–4-fold increase in cytochrome C content was found in the cytosol of all three colon cancer cell lines exposed to MPE treatment, and such an effect occurred concomitantly to its reduction in the mitochondrial fraction. This result was more evident in both Caco-2 and HCT116 treated cells.

Interestingly, under the same experimental conditions, an increase in AIF levels was also observed in the cytosolic fraction, indicating that MPE causes the release of mitochondrial apoptogenic factors—probably as a consequence of the mitochondrial membrane potential dissipation. 

### 2.5. Analysis of the Cytotoxic Effects Exerted by Phytochemicals Contained in MPE

**Figure 6:**
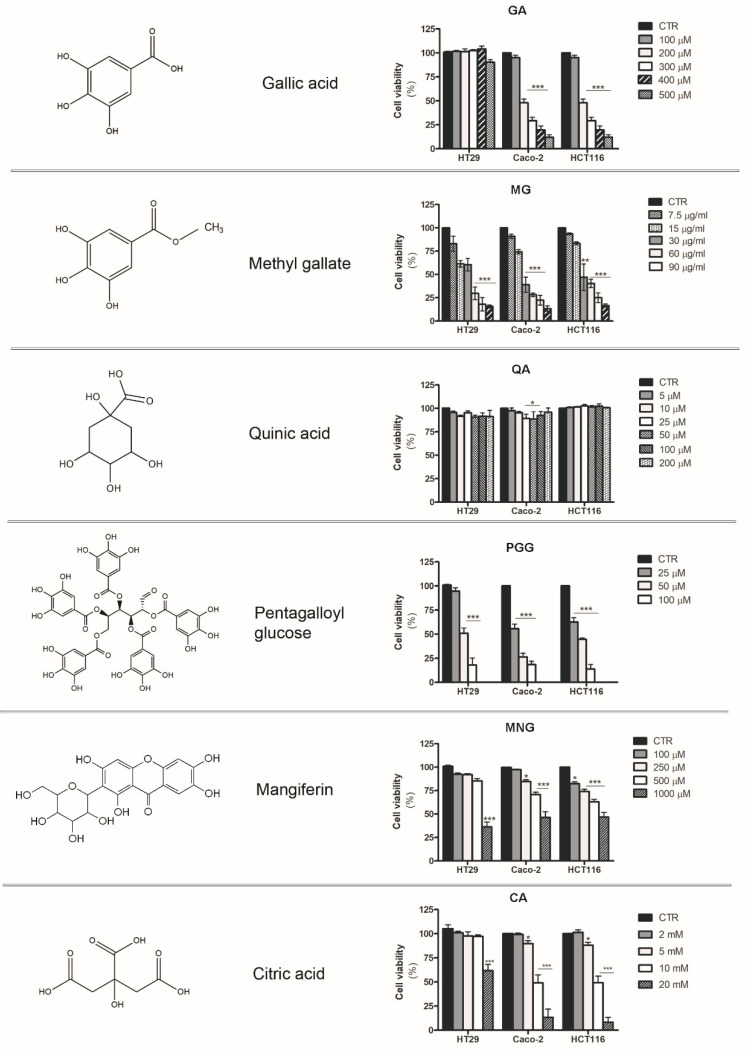
Cell viability of colon carcinoma cells treated with phytocompounds contained in MPE. Colon cancer cell lines were incubated in the presence of different doses of the main phytochemicals (GA: gallic acid; MG: methyl gallate; QA: quinic acid; PGG: pentagalloyl glucose; MNG: mangiferin; and CA: citric acid) contained in MPE for 48 h. Chemical structures of phytocompounds reported in the left panel were drawn using ChemDraw software. The percentage of viable cells was evaluated by MTT assay, as reported in the Materials and Methods section. In the figure, data are reported in a bar chart, and are the means of three independent experiments ± SD. Statistical significance was assessed using Student’s *t*-test: (*) *p* < 0.05, (**) *p* < 0.01, and (***) *p* < 0.001 versus untreated control.

Our previous studies provided evidence that the peel fraction of mango fruit is rich in gallic acid (GA), mangiferin (MNG), citric acid (CA), quinic acid (QA), and esters of gallic and digallic acids—such as methyl gallate (MG) and pentagalloyl glucose (PGG), which were the most represented bioactive compounds [21]. In order to clarify the molecules responsible for the antitumor action of MPE, we tested the effects of single phytoconstituents present in the crude extract. As shown in Figure 6, the biological activity of MPE observed in colon cancer cells could be ascribed to gallic acid, mangiferin, pentagalloyl glucose, and methyl gallate, which were capable of markedly reducing cell viability in all three colon cancer cell lines in a dose-dependent manner after 48 h of exposure. As reported in the figure, an inhibitory effect of about 50% was observed with 200 μM GA and 10 mM CA in both Caco-2 and HCT116 cells, while they were scarcely effective in HT29 cells. 

The most significant effect was observed in all three colon cancer cells with pentagalloyl glucose and methyl gallate, which exerted a potent growth inhibition on the cancer cells tested (Figure 6).

Taken together, these results highlight the ability of some bioactive compounds found in fruit peel to inhibit cell viability, providing evidence that some esters of gallic acid were the most active. Additional studies are needed in the future in order to better define their mode of action.

## 3. Discussion

In recent years, a mounting interest has emerged in the role and mechanisms played by nutrient supplements and bioactive components as natural compounds that should be included in everyone’s diets for their contributions to people’s wellbeing [29,30]. This interest is also supported by epidemiological studies that demonstrate the value of nutrients in inhibiting cancer growth, thus offering chemopreventive action. Such observations have prompted even more people to adopt a healthier food regimen. On the other hand, compelling evidence has also noted how, among phytochemicals, polyphenols—including phenolic acids, flavonoids, stilbenes, and lignans—play essential roles in human health, providing valuable benefits [31]. Indeed, these secondary metabolites of plants have been proven to modulate different cell signaling pathways correlated with cell cycle control [32], cell demise programs such as apoptosis and autophagy [33], and inflammatory processes [34], which puts them in the spotlight for cancer research for their antitumor profiles. 

In this scenario, a plethora of beneficial effects have been described for *Mangifera indica L.* fruit preparations, which possess a valid nutraceutical potential for anticancer, immunomodulatory, radioprotective, antidiabetic, anti-inflammatory, and antioxidant properties [12,16,35,36,37,38]. 

The antitumor ability of crude extracts of mango fruit has been largely explored. The edible part of the fruit, known as pulp, tested in in vitro models represented by a colon adenocarcinoma cell line (SW480), as well as in mice with colon carcinoma, exerted a prominent antiproliferative activity [39]. Similar effects were also described for other parts of the fruit, such as the seed, which significantly reduced the cell viability of SW40 and COLO 320DM colon carcinoma cells and HeLa cervical cancer cells, while being ineffective on normal human lymphocytes [40]. 

Although the beneficial properties of mango have been well described, it is worthy of note that the chemical composition of mango, as well as the associated biological activity, can vary in relation to the area of cultivation, and with the ripeness of the fruit at the time of harvest. All of these conditions can seriously affect both organoleptic features and phytocompound profiles, offering different attributes that meet consumers’ appreciation [15].

In recent years, *Mangifera indica L.* crops have been introduced in Sicily (Italy), where the favorable climate offers the opportunity to obtain fresh products rich in the phytochemicals that have increased consumer demand. Our previous investigations specifically focused on Sicilian mango fruit demonstrated that mango peel extract (MPE) can exert remarkable cytotoxic effects on human colon cancer cells, while only modest effects occur in normal human fibroblasts [21]. The antitumor action was the consequence of programmed apoptotic cell death associated with precocious ROS generation, DNA fragmentation, PARP cleavage, and a general genotoxic stress evidenced by H2Ax phosphorylation [21]. The data reported here provide evidence that MPE induced a rapid generation of mitochondrial superoxide anions, associated with a consistent thiol group decline that could be at the root of mitochondrial dysfunction triggered by MPE. Such an experimental observation was supported by the dissipation of Δψm, with the consequent mitochondrial release of cytochrome C and AIF—two well-known apoptogenic factors. AIF is an apoptosis-promoting factor that can be released from the mitochondria following the loss of Δψm and redistributed into the nuclear compartment to take part in chromatin condensation and DNA fragmentation [41]. In agreement with these data, our preliminary results seem to indicate that MPE treatment can promote the nuclear localization of AIF. However, considering the pleiotropic role of this factor in cell death mechanisms, we intend to deepen the analysis of AIF at the nuclear level in our future studies.

Under our experimental conditions, the mitochondrial dysfunction was also sustained by the drop of VDAC1, as well as mitofilin—a mitochondrial membrane protein involved in the shaping and remodeling of the cristae. These data were consistent with a study by Madungwe et al. [42] demonstrating that mitofilin knockdown is necessary in order to trigger apoptosis via the activation of the AIF–PARP pathway. 

Altogether, our findings seem to indicate that the loss of redox balance, mediated by both genotoxic stress and mitochondrial injury, may represent the causative events orchestrating the antitumor action of MPE. The same treatment caused the mitochondrial release of cytochrome C, whose translocation could irretrievably concur with the apoptotic programmed cell death that we demonstrated in our previous studies. Cytochrome C can indeed participate in the cell death process upon its association with apoptotic protease-activating factor-1 (Apaf-1) to form the apoptosome, or localizing into the nucleus to support the molecular repair machines at DNA-damaged sites induced by caspase-independent events. [43,44]. However, we cannot exclude the possibility that cytochrome C release is related to mitofilin downregulation promoted by MPE treatment. This hypothesis is sustained by Yang’s studies demonstrating that mitofilin knockdown makes cells more prone to intrinsic apoptotic death, causing the release of cytochrome C from the mitochondrial cristae [45].

Altogether, our results indicate that MPE induces a canonical apoptotic pathway that likely starts in the mitochondria.

In light of our previous data concerning the chemical characterization of MPE [21], we also demonstrate that gallate esters—the most representative constituents of MPE—could be responsible for the observed anticancer effects. However, despite the findings reported here, further in-depth analyses will be needed in future in order to better understand whether MPE cytotoxicity can be ascribed to a specific compound, or to a combination of two or more phytochemicals that could exert their antitumor potential by acting in a synergistic manner in the extract. 

## 4. Materials and Methods

### 4.1. Cell Cultures and Chemicals

Human adenocarcinoma HT29, Caco-2, and carcinoma HCT116 cells (Interlab Cell Line Collection, ICLC, Genoa, Italy) were cultured in complete RPMI medium (Euroclone, Pero, Milan) supplemented with 10% heat-inactivated FBS (Life Technologies, Milan, Italy), 2 mM l-glutamine, streptomycin (100 U/mL), penicillin (100 U/mL), and 1% nonessential amino acids (Biowest, MO, USA) in a humidified incubator at 37 °C and with a 5% CO_2_ atmosphere. After seeding on 96- or 6-well plates, cells were incubated overnight in order to allow adhesion, and when reaching a 70% confluence they were treated in the presence of mango extracts, phytocompounds, or vehicle only as described below. The final concentration of ethanol or DMSO employed as a vehicle had no noticeable effects on the treated cells in comparison to control cells.

The phytochemicals—such as gallic acid, citric acid, methyl gallate, penta-O-galloyl-β-d-glucose hydrate, quinic acid, and mangiferin—as well as the other reagents and compounds, were purchased from Sigma-Aldrich (Milan, Italy), except where stated otherwise.

### 4.2. Preparation of Mangifera Indica Peel Extracts

Initially, mango fruits were washed, and their peel was cut into small pieces and lyophilized for one night using a Hetosicc Heto lyophilizer (Hetosicc, Heto, Birkerød Denmark). Then, a fine powder was obtained from the pieces previously lyophilized, using a stainless steel grinder. The powders were successively solubilized in a hydro-alcoholic solution (1:1 ethanol/PBS), to a final concentration of 75 mg/mL, and kept overnight at 37 °C in constant agitation. Next, the extract was firstly centrifuged at 120× *g* for 10 min, and then the supernatant was exposed to a second centrifugation at 15,500× *g* for an additional 10 min. The final extract, represented by the supernatant, was then stored at 20 °C until use for experimental assays. 

The experiments were performed with a dose of MPE (360 μg/mL) that demonstrated good efficacy in our previous studies [21]. All working solutions used for the treatments were diluted in cell culture medium. 

### 4.3. Analysis of Cell Viability by MTT Assay

The MTT (3-(4′,5′-dimethylthiazol-2′-yl)-2,5-diphenyltetrazolium bromide) colorimetric assay was employed to evaluate the effects of the different phytocompounds found in MPE. For these experiments, 8 × 10^3^ colon cancer cells were seeded in a 96-well plate and exposed to the treatment, as reported in the Results section. Then, the assay was performed, as we reported previously [46]. The optical density (OD) of the formazan produced by viable cells was measured with an ELISA microplate reader (OPSYS MR, Dynex Technologies, Chantilly, VA, USA) at 490 nm, using 630 nm as a reference wavelength. Cell viability values were indicated as percentages and were calculated using the following formula: ((OD sample/OD control) × 100). Reported data are the result of three experiments performed in triplicate.

### 4.4. Analysis of Thiol Group Content 

The thiol group concentration was assayed using Ellman’s reagent method [47]. To measure intracellular protein thiols, cells were plated in 10-mm dishes at a cell density of 1 × 10^6^ cells/dish. After the designed MPE treatment, cells were detached via trypsinization, washed in PBS, and resuspended in 30 mM Tris–HCl, 3 mM EDTA, pH 8.2. Cellular proteins were precipitated by adding 25 μL of 1.5 mM 5.5’-dithiobis-(2-nitrobenzoic acid) (DTNB, Ellman’s reagent) and 400 μL of methanol to aliquots. The mixture was then centrifuged at 3000× *g* for 5 min, and then 250 μL of each supernatant was transferred into a 96-well plate to be read at 412 nm using an ELISA plate reader (OPSYS MR, Dynex Technologies, Chantilly, VA, USA).

In order to analyze the free thiol groups, cells were resuspended in 10% trichloroacetic acid (TCA) and centrifuged. Subsequently, 50 μL of each supernatant was transferred into a 96-well plate, adding 200 μL 0.2 M Tris–HCl, pH 8.9, and 20 μL DTNB. The final content of intracellular protein thiols was determined by measuring the absorbance at 412 nm. The results obtained from the analysis of thiol group content were normalized to protein content and expressed as nanomoles of SH groups per 10^5^ cells, using a GSH standard curve.

### 4.5. Detection of Mitochondrial Superoxide Anion Production

The production of mitochondrial superoxide anion was assessed using MitoSOX Red reagent (Thermo Fisher Scientific, Monza, Italy)—a live-cell permeable dye that selectively targets mitochondrial superoxides, producing red fluorescence after oxidation. Cells (8 × 10^3^/well) were treated with MPE, then incubated for 10 min in the presence of 5 μM MitoSOX Red solution prepared in HBSS/Ca/Mg, according to the manufacturer’s instructions. Cells incubated in the presence of rotenone were used as positive controls for superoxide production. The positive cells were visualized on a Leica fluorescence microscope with a rhodamine filter (excitation wavelength of 596 nm and emission wavelength of 620 nm). The images were taken using Leica Q Fluoro Software, and the quantitative analysis of fluorescence levels was performed using ImageJ software.

### 4.6. Analysis of Mitochondrial Membrane Potential

To evaluate the effect of MPE on mitochondrial membrane potential (Δψm), HT29, Caco-2, and HCT116 cells (8 × 10^3^) were seeded in a 96-well plate. After exposure to MPE, cells were incubated for 15 min with JC-1 staining solution [48], and Δψm was assessed according to the protocol provided by the Cayman Chemical Company (Ann Arbor, MI, USA). JC-1 is a specific potentiometric dye that enters the mitochondria, producing a different staining of healthy and unhealthy cells. The compound forms aggregates with intense red–orange fluorescence, detectable with a rhodamine filter (excitation wavelength of 596 nm and emission wavelength of 620 nm) in healthy cells with polarized mitochondria and high Δψm. Conversely, JC-1 can remain in a monomeric form, showing green fluorescence detectable with an FITC filter (excitation wavelength of 485 nm and emission wavelength of 530 nm) in unhealthy cells that exhibit low Δψm. All images were visualized using a Leica fluorescence microscope (Leica Microsystems) equipped with a DC300F camera. The merging of images taken with the two filters (rhodamine vs. FITC) was performed using the Leica Q Fluoro Software (Wetzlar, Germany). We selected a minimum of three fields for each condition in order to calculate the JC-1 aggregates/JC-1 monomers ratio. The reduction of this ratio was considered to signify loss of Δψm.

### 4.7. Protein Extraction and Western Blot Analysis

Proteins were analyzed by Western blot. Briefly, after treatment with MPE, the preparation of cell lysates was conducted, as previously reported [49]. Subsequently, equivalent amounts of protein sample (30 μg) were resolved using SDS-PAGE and electroblotted on a nitrocellulose membrane filter (Bio-Rad Laboratories Srl). Then, membranes were blocked with 1–5% non-fat dry milk and incubated with primary antibodies overnight at 4 °C. Except for cytochrome C, which was purchased from Cell Signaling Technology (Beverly, MA, USA), and mitofilin, which was provided by Novus Biologicals (Littleton, CO, USA), all other antibodies (Bcl-2, Bcl-X_L_, Mcl-1, COX IV, MnSOD, VDAC1, AIF) were provided by Santa Cruz Biotechnology (Santa Cruz, CA, USA). Detection was completed using secondary antibodies (1 h at 4 °C) conjugated with horseradish peroxidase (HRP) (rabbit or mouse, 1:5000) obtained from Amersham, GE Healthcare Life Science (Milan, Italy). Protein bands were detected via Westar Ultra 2.0 enhanced chemiluminescence (ECL) reagent (Cyanagen, Bologna, Italy) using the ChemiDoc XRS System (Bio-Rad, Hercules, CA, USA). Densitometric quantification of bands was performed using Quantity One software (Bio-Rad). γ–Tubulin (diluted 1:1000; Sigma-Aldrich, Milan, Italy) was employed as an internal loading control for normalization, after ensuring that its expression was not modified under different experimental conditions.

### 4.8. Preparation of Mitochondrial and Cytosolic Fractions 

Subcellular fractionation to obtain cytosolic and mitochondrial compartments was performed according to the procedure described by Dimauro et al. [50]. After treatment with MPE, cells (1.5 × 10^7^) were washed twice in PBS, resuspended in STM Buffer (250 mM sucrose, 50 mM Tris–HCl pH 7.4, 5 mM MgCl_2_, and protease inhibitor cocktail), homogenized with a Teflon homogenizer, and passed through a 25-gauge needle 30–40 times (step repeated twice). Next, the homogenates were placed on ice for 30 min, vortexed for 15 s, and centrifuged at 800× *g* for 15 min at 4 °C in order to obtain the supernatant (S0) used to isolate the mitochondrial and cytosolic fractions, while Pellet P0 was discarded.

Next, the supernatant S0 was centrifuged at 800× *g* for 10 min. Pellet P2 was discarded, while supernatant S2 was further centrifuged for 15 min at 15,500× *g* for the preparation of Supernatant S3 and Pellet P3. Pellet P3 was resuspended in STM buffer and centrifuged at 13,000× *g* for 10 min in order to obtain pellet P4, which was resuspended in SOL buffer (50 mM Tris HCl pH 6.8, 1 mM EDTA, 0.5% Triton-X-100, and protease inhibitors), and represented the mitochondrial fraction (Supernatant S4 was discarded). Conversely, Supernatant S3 was precipitated by incubation with 100% cold acetone (1:1 volume) for 2–3 h at −20 °C, and then was centrifuged at 13,000× *g* for 5 min. The resulting pellet was diluted in STM buffer, and represented the cytosolic fraction. 

Thirty micrograms of proteins of the mitochondrial and cytosolic fractions were analyzed via Western blot analysis in order to evaluate cytochrome C and AIF content. COX IV and γ–tubulin were used as mitochondrial and cytosolic loading markers, respectively.

### 4.9. Statistical Analysis

Data were analyzed statistically and reported as the means ± SD (standard deviation). Student’s *t*-test was used for statistical comparison between control (untreated) and treated groups. Data analysis was accomplished using the GraphPad Prism 5.0 software package (San Diego, CA, USA). Differences were significant when *p* < 0.05. 

## Data Availability

Data are contained within the article.

## Sample Availability

Samples of the compounds are not available from the authors.
